# The Spectra of Disease-Causing Mutations in the Ferroportin 1 (*SLC40A1*) Encoding Gene and Related Iron Overload Phenotypes (Hemochromatosis Type 4 and Ferroportin Disease)

**DOI:** 10.1155/2023/5162256

**Published:** 2023-06-13

**Authors:** Kevin Uguen, Chandran Ka, Gwenaelle Collod-Béroud, Marlène Le Tertre, Julie Guellec, Claude Férec, Christophe Béroud, Isabelle Callebaut, Gérald Le Gac

**Affiliations:** ^1^Univ Brest, Inserm, EFS, UMR1078, GGB F-29200, France; ^2^CHRU de Brest, Service de Génétique Médicale et Biologie de la Reproduction, Laboratoire de Génétique Moléculaire et Histocompatibilité, F-29200, France; ^3^Laboratory of Excellence GR-Ex, F-75015, France; ^4^Aix Marseille University, INSERM, Marseille Medical Genetics, F-13005, France; ^5^Association Gaétan Saleün, F-29200, France; ^6^Sorbonne Université, Muséum National d'Histoire Naturelle, UMR CNRS 7590, Institut de Minéralogie, de Physique des Matériaux et de Cosmochimie, IMPMC, F-75005, France

## Abstract

SLC40A1 is the sole iron export protein reported in mammals and is a key player in both cellular and systemic iron homeostasis. This unique iron exporter, which belongs to the major facilitator superfamily, is predominantly regulated by the hyposideremic hormone hepcidin. SLC40A1 dysfunction causes ferroportin disease, and autosomal dominant iron overload disorder characterized by cellular iron retention, principally in reticuloendothelial cells, correlating with high serum ferritin and low to normal transferrin saturation. Resistant to hepcidin, SLC40A1 mutations are rather associated with elevated plasma iron and parenchymal iron deposition, a condition that resembles *HFE*-related hemochromatosis and is associated with more clinical complications. With very few exceptions, only missense variations are reported at the *SLC40A1* locus; this situation increasingly limits the establishment of pathogenicity. In this mutation update, we provide a comprehensive review of all the pathogenic or likely pathogenic variants, variants of unknown significance, and benign or likely benign *SLC40A1* variants. The classification is essentially determined using functional, structural, segregation, and recurrence data. We furnish new information on genotype-phenotype correlations for loss-of-function, gain-of-function, and other *SLC40A1* variants, confirming the existence of wide clinical heterogeneity and the potential for misdiagnosis. All information is recorded in a locus-specific online database.

## 1. Introduction

The solute carrier family 40 member 1 (SLC40A1), now preferably referred to as ferroportin 1 (FPN1), is the sole iron export protein reported in mammals and is a key player in both cellular and systemic iron homeostasis [1–3]. It is expressed in all types of cells that handle major iron flow, including macrophages, duodenal enterocytes, hepatocytes, erythrocytes, and placenta syncytiotrophoblasts [4–6]. SLC40A1 activity is predominantly regulated by the liver-derived peptide hepcidin which, depending on the cell type, induces internalization and degradation of SLC40A1 or blocks the SLC40A1-related iron transport mechanism (occlusive effect of hepcidin), thus decreasing iron delivery to plasma [5].

The *SLC40A1* gene (LRG_837), which is located on chromosome 2 (2q32.2), is composed of eight coding exons that are spread on approximately 20 kb of genomic DNA and give rise to a 571-amino acid protein of 62.5 kDa (UniProtKB: Q9NP59). The reference transcript (ENST00000261024.6; NM_145585.5) contains an iron-responsive element (IRE) in its 5′-untranslated region (UTR), which allows for posttranscriptional regulation via the iron-responsive element/iron regulatory protein (IRE/IRP) system as observed for other important iron metabolism genes [7]. Under low-iron conditions, IRPs bind to the 5′-UTR IRE, resulting in inhibition of FPN1 translation and reduction of iron efflux from cells. It is noteworthy that duodenal epithelial and erythroid precursor cells express a shortened *SLC40A1* transcript (in addition to the canonical one), which encodes the full-length protein but lacks the IRE and thus escapes translational repression [8].

SLC40A1 belongs to the major facilitator superfamily (MFS), which is the largest group of phylogenetically related secondary active transporters (commonly called “solute carriers,” SLCs). MFS proteins are present in cells across all life kingdoms, controlling the flow of a wide range of substrates (inorganic ions, metabolites, neurotransmitters, and drugs) over lipid bilayers [9, 10]. Despite low sequence similarities, MFS members share a common architecture consisting in twelve transmembrane (TM) helices organized into two structurally similar lobes, each including 6 TMs (N-lobe: TM1-TM6 and C-lobe: TM7-TM12). The two lobes interact together and orchestrate transitions between two extreme conformational states, outward-facing (OF) and inward-facing (IF), according to the alternating access model of membrane transport [11, 12]. During the structural transitions, the substrate sits at the approximate center of the transporter and is not accessible to either side of the membrane.

The existence of an adult, autosomal dominant primary iron overload, soon regarded as an atypical form of hemochromatosis (HC) and now also referred to as ferroportin disease (FD) [13], was based on the description of two *SLC40A1* missense mutations, namely, p.Ala77Asp and p.Asn144His, in Italian and Dutch pedigrees [14, 15]. The study of few other families [16] and the achievement of *in vitro* assays [17, 18] then revealed the existence of two functional categories of *SLC40A1* gene mutations underlying two distinct clinical entities. Loss-of-function (LOF) mutations, such as p.Ala77Asp, affect cellular localization of SLC40A1 and/or its iron export function. This results in cellular iron retention, principally in reticuloendothelial cells, and relative plasma iron deficiency. Laboratory tests show a significant increase in serum ferritin, which contrasts with a normal or slightly increased transferrin saturation. As the disease progresses, iron is deposited in parenchymal cells, normally accompanied by a rise in transferrin saturation. This iron overload phenotype, which has historically been associated with HC type 4A, is typical of FD. Gain-of-function (GOF) mutations, such as p.Asp144His, result in partial to complete resistance to hepcidin. This leads to excessive iron export to the bloodstream, increased transferrin saturation, and progressive iron accumulation in parenchymal cells (primarily hepatocytes). These biologic and histologic features mimic the natural history of *HFE*-related HC; hence, GOF *SLC40A1* mutations are associated with HC type 4 (historically referred to as HC type 4B and nowadays also described as nonclassical FD) [19, 20].

The interpretation of missense variations and their association with disease phenotypes is one of the greatest challenges in medical genetics. This is particularly true for *SLC40A1*-related diseases, which are overwhelmingly associated with rare or private missense variations and are characterized by incomplete penetrance and variable expressivity, with several phenocopies that may confound clinical diagnosis [20]. Pedigree analyses have revealed the influence of age and sex in the progression of iron overload, with clinical phenotypes being mainly observed in adult males [21]. Phenocopy, defined as an environmentally induced, nonhereditary phenotype that mimics one produced by a gene [22], is a growing concern due to increasing descriptions of variants in isolated individuals with hyperferritinemia. *In vitro* assays are increasingly used to differentiate causal mutations from neutral variations identified in suspected FD or HC type 4 patients. Some experimental data are however questionable, with no obvious relationship with clinical observations [20]. Various *in silico* prediction tools have also been tested but have not yet been found to be very informative [20, 23], probably due to the nonconsideration of the molecular-level effect of missense variants in the context of SLC40A1 3D structures. The problem may be exacerbated by the existence of several distinct disease mechanisms, some of which combine loss and gain of FPN1 function and/or depend on specific groups of amino acids [24, 25].

In this study, we provide an overview of all *SLC40A1* variants that were published before January 2022. *SLC40A1* variants associated with relevant phenotypical data were manually curated and entered into a new UMD locus specific database at http://umd.be/SLC40A1/. We summarize the latest *in vitro* data and provide a structural rationalization of LOF and GOF mutations. We classify all the curated variants according to the American College of Medical Genetics (ACMG) and Association for Molecular Pathology (AMP) guidelines and finally discuss the biological effect and clinical relevance of pathogenic or likely pathogenic mutations and variants of unknown significance.

## 2. Variants

**Figure 1:**
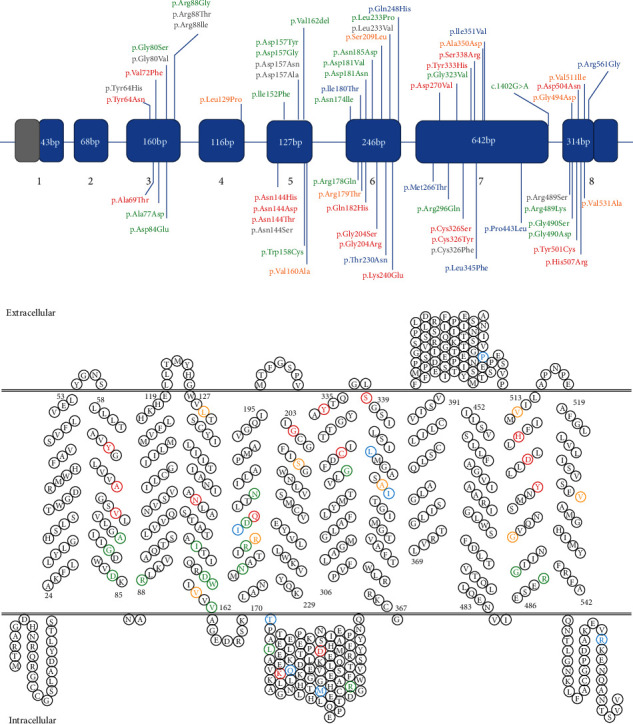
Distribution of the 65 selected variants along the *SLC40A1* gene and the secondary structure of the SLC40A1 protein. (a) The *SLC40A1* sequence is represented as a horizontal bar, and each of the segments corresponds to individual exons (coding parts in blue and 5′- and 3′-untranslated regions in grey), in proportional size and numbered. Introns are represented as a short nonproportional line linking exons. Banners above or below the coding sequence indicate the positions of all variations included in Table 1. Variations that have been studied *in vitro* are presented in green (loss-of-function), red (gain-of-function), or blue (no functional impact). Missense variations with no functional evidence of deleteriousness or neutrality are presented in grey (for 10 substitutions that occur at the same 8 positions than other variations described as LoF or GoF) or orange (for 8 substitutions that occur at 8 positions where no well-recognized pathogenic variant has been seen to date). (b) The 48 amino acid positions known to be mutated in patients with various iron overload phenotypes reported on the secondary structure topology of the human SLC40A1; amino acids for which variations are associated with a functional defect are colored in green (LoF) or red (GoF), whereas those for which no substitution has been characterized functionally are colored in orange. The human FPN1 protein (UniProtKB: Q9NP59) is made of 571 amino acids.

Each published variation was checked for accuracy and named, or curated, using the Human Genome Variation Society nomenclature (https://varnomen.hgvs.org/) and the canonical full-length reference transcript NM_014585.5 (in human genome GRCh37). Of the total 65 variants compiled in Table 1 (corresponding to 48 different amino acids; Figure 1), 40 were found in single patients or single families (62%), 21 were recurrent in up to four families (32%), and only four (p.Arg178Gln and p.Gly490Asp, p.Gly80Ser, and p.Val162del) were reported in nine, 12, and 20 families, respectively (6%).

It is worth mentioning that none of the heterozygous variants currently associated with *SLC40A1*-related iron overload phenotypes appear to result in a total absence of iron export. This is certainly due to the very important and unique function of the SLC40A1 protein, which is essential for placental iron transport and early embryonic development, dietary iron absorption, cellular iron homeostasis, and iron distribution between tissues [1–3]. The strength of selection acting on heterozygotes is so high (as also indicated by the pLI and LOEUF gnomAD-derived constraint scores: 0.99 and 0.29, respectively [26]) that the missense variation type is almost exclusive (98%). Apart from this type of variation, only the p.Val162 single-amino-acid in-frame deletion has been consistently associated with iron overload phenotypes. The p.Gly468Ser substitution can be considered separately, in that the underlying c.1402G>A transition disrupts the exon 7 donor site, resulting in the partial use of an exonic cryptic splice site and the generation of a truncated reading frame [27, 28], while the Gly468 to Ser amino acid change by itself has no obvious effect on the ability of SLC40A1 to export iron [27].

### 2.1. Functional Annotation of *SLC40A1* Variants according to *In Vitro* Analyses

The available experimental information, taken from 28 publications and a variety of heterologous cell-based assays (Table 1), was manually curated and used for *SLC40A1* variant classification into “loss-of-function” (LoF), “gain-of-function” (GoF), or “neutral” categories. Twenty-one (of 65) *SLC40A1* variants that were proven to reduce cell surface expression and/or result in decreased iron export (impact on FPN1 biogenesis or its conformational changes within the lipid bilayer) were classified as LoF (colored in green in Figure 1(a)). Eighteen *SLC40A1* variants that did not significantly alter cell surface expression and iron export, but were shown to confer total or partial resistance to hepcidin, were classified as GoF (colored in red in Figure 1(a)). Eight *SLC40A1* variants that produced no obvious change from the wild-type protein were listed as “neutral” (colored in blue in Figure 1(a)). It is important to mention that the eight variants were also investigated for their possible effect on pre-mRNA splicing and that no abnormalities were found [29].

No functional data was available for 17 missense variants, while we decided to ignore those published for the p.Ser209Leu substitution because of a substantial discrepancy between the reported biological data in 7 patients, only suggesting FD [30, 31], and the assumption made by An P. and collaborators from *in vitro* studies of a gain-of-function mechanism (without evidence of altered iron export) [30].

Ten of the eighteen missense variants without functional data were at the same eight positions (Tyr64, Gly80, Arg88, Asn144, Asp157, Leu233, Cys326, and Arg329) than substitutions that were analyzed *in vitro* and recognized as either LoF or GoF mutations (colored in grey in Figure 1(a)). Five of the eight residues thus considered were found to be strictly conserved in all vertebrates, whereas the remaining three (Asn144, Cys326, and Leu233) diverged only in bacteria (Supplementary Figure 1). Some interesting differences were observed between amino acid pairs in terms of side chain atom composition, polarity, and volume by using the Grantham matrix [32, 33] (Supplementary Table 1), but they were not considered sufficient to predict the deleteriousness of the variants (also see Section 2.3).

The functional importance of Leu129, Val160, Arg179, Ser209, Ala350, Gly494, Val511, and Val531 (colored in orange in Figure 1(a)) is yet to be elucidated. The eight residues are relatively well conserved in vertebrates (Supplementary Figure 1), suggesting the existence of structural and/or functional constraints, but they occur in regions of the protein whose functional importance has not yet been specified. The cellular effects of changes at these positions thus remain elusive.

### 2.2. *SLC40A1* Variant Spectrum and Structural Rationalization of LoF and GoF Mutations

As illustrated in Figure 1(a), the 65 variants investigated in this study have been identified throughout the *SLC40A1* coding sequence, with the exception of exons 1 and 2. No hotspot can be clearly defined, although ten residues are found to be recurrently mutated: p.Tyr64, p.Gly80, p.Arg88, p.Asn144n, p.Asp157, p.Gly204, p.Leu233, p.Cys326, p.Arg489, and p.Gly490. Functionally characterized LoF (variants in green) or GoF (variants in red) has been reported at any of these 10 amino acid positions. Similarly, variants have been identified in various topological regions of the SLC40A1 protein, with the exception of the N-terminus and the transmembrane helices 1, 9, and 10 (Figure 1(b)).

**Figure 2:**
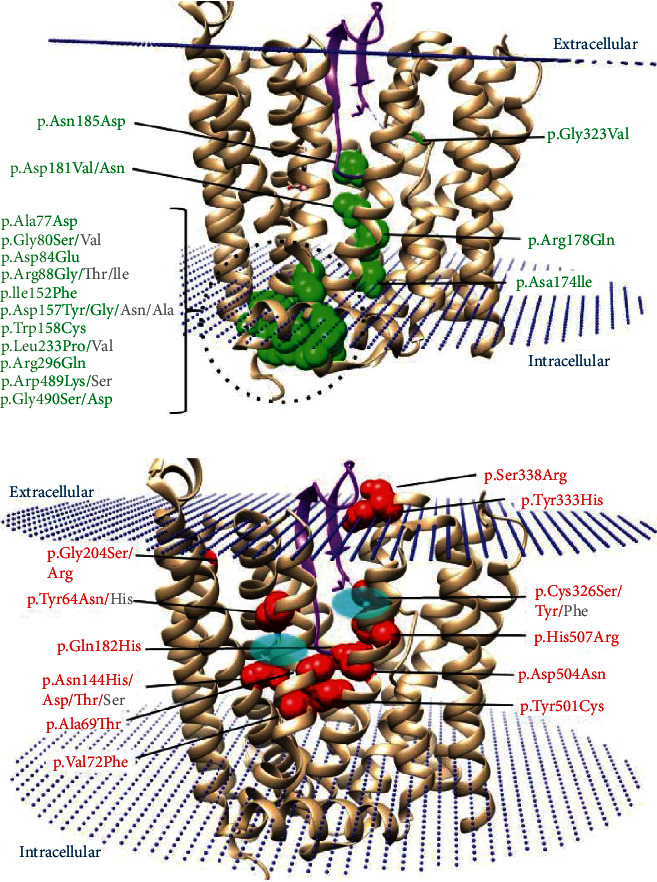
Ribbon representation of the 3D structure of human SLC40A1 in complex with hepcidin (pdb 6WBV), highlighting the distribution of the loss-of-function mutations (a) and gain-of-function mutations (b). The hepcidin hyposideremic hormone is colored in magenta. Amino acids for which mutations are reported as LoF are colored in green, whereas those for which mutations are reported as GoF are colored in red. Banners indicate the considered amino acid changes and their positions within the protein. The positions of the two divalent metal-binding sites within the central cavity are delimited by blue ellipses. The protein was positioned in a lipid bilayer using the PPM web server [75].

Billesbølle et al. recently used cryoelectron microscopy to solve three-dimensional structures of human SLC40A1 (HsSLC40A1) in the outward-facing conformational state, which is regarded as the basal state [34]. The mapping of disease-causing mutations on the HsSLC40A1 3D structure with hepcidin (pdb 6WBV) leads to observe a clear separate distribution into functional subtypes (Figure 2).

The loss-of-function missense mutations are rather located at the cytoplasmic interface (Figure 2(a)) suggesting that this region may be important for the iron export function. This point was already made by Wallace et al. over 10 years ago [35], based on *de novo* 3D structure models. As a matter of fact, we have shown that the intracellular gate, which regulates access from the cytoplasm to the central substrate-binding pocket (or central cavity), is stabilized by noncovalent interactions (salt bridges and hydrogen bonds) between the TM2, TM3, TM4, and TM11 transmembrane helices, on the one hand, and the TM5 and TM10 transmembrane helices, on the other hand [25]. We identified 12 residues (Asp81, Asp84, Lys85, Arg88, Asp157, Asn174, Arg178, Asp473, Gln478, Arg489, Gly490, and Ile491) that are critical for protein stability and/or conformational changes during the iron transport cycle. We pointed out that a total of 13 missense variations affecting 7 (Asp84, Arg88, Asp157, Asn174, Arg178, and Gly490) of the 12 considered gating residues were reported in 77 patients with common features of FD. These missense variations could be responsible for two distinct loss-of-function mechanisms: protein misfolding and reduced expression on the cell surface, or failure to export iron [25, 36, 37].

The gain-of-function mutations are rather concentrated around the central cavity (Figure 2(b)), which actually contains two divalent metal-binding sites (one in each lobe) and the hepcidin-binding site [34]. This last one is centered on the Cys326 residue, which is strictly conserved in all vertebrates and may have evolved in conjunction with hepcidin to control systemic iron homeostasis [38]. At first glance, a functional dichotomy seemed to emerge between missense mutations located in the C-lobe, in particular those affecting positions Cys326, Asp504, and His507, which were associated with the highest degree of hepcidin resistance, and missense mutations located in the N-lobe, in particular those affecting the recurrently mutated Gly204 and Asn144 positions, which were found to induce partial resistance to hepcidin [39]. But things could actually be more complex and depend on the effect of mutations on hepcidin binding (on each lobe) and/or hepcidin-dependent ubiquitination (in the case of hepcidin-induced endocytosis and degradation of SLC40A1) following subtle conformational changes [24, 34]. The effects of different mutations may also vary depending on the cell type [24].

Given the proximity between amino acids participating in the binding of iron or hepcidin and the involvement of some gating residues in conformational changes also governing the iron export function and its regulation by hepcidin, one can anticipate the existence of a third class of SLC40A1 pathogenic variants with both loss- and gain-of-function features. Examples supported by *in vitro* studies exist in the literature, but they are still very limited [24, 40, 41].

### 2.3. Structural Annotation of *SLC40A1* Missense Variants without Functional Data

To better predict effects of the *SCL40A1* missense variants occurring at recurrently mutated positions, especially those that are more likely to be tolerated (substitution with chemical similar amino acids), we leveraged the 3D structure of human SLC40A1 (in its outward-open facing conformation) taking into account current knowledge about the iron export mechanism and its regulation by hepcidin.

As aforesaid, Arg88, Asp157, and Arg489 play a critical role in the architecture of the human SLC40A1 intracellular gate [25]. The loss of a salt bridge in the cytoplasmic network connecting these three charged amino acids can result in folding defects in TM helices TM3, TM4, or TM11 and their assembly or local instability of the SLC40A1 structure in its outward-open conformation. In this regard, it is very likely that the p.Arg88Thr, p.Arg88Ile, p.Asp157Asn, p.Asp157Ala, and p.Arg489Ser missense variants reduce ability of SLC40A1 to export iron out of cells.

**Figure 3:**
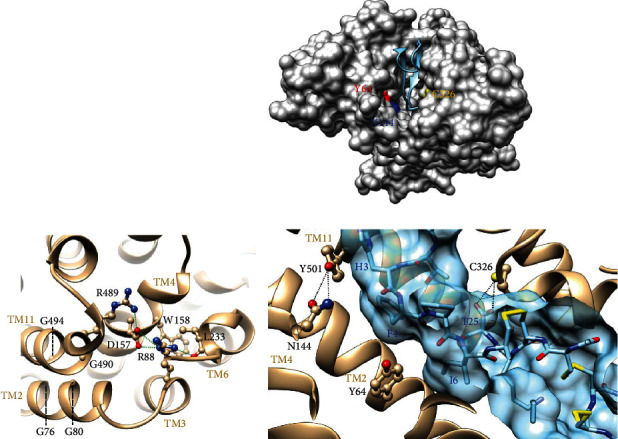
Close-up views of the 3D structure of human SLC40A1 (pdb 6WBV). (a) Ribbon representation of the inner gate in which amino acids discussed in Section 2.3 are highlighted in atomic details. (b) Top: solvent accessible surface of SLC40A1, with the bound hepcidin displayed in a ribbon representation. Tyr64, Asn144, and Cys326 are colored red (oxygen atom), blue (nitrogen atom), and yellow (sulfur atom), respectively. Bottom: focus on Tyr64, Asn144, and Cys326. Hepcidin is shown in blue.

As shown in Figure 3(a), some other 3D structure features are likely to contribute to the stability of the human SLC40A1 inner gate. First, a tight packing of the helices TM2 and TM11 is ensured by two glycine zipper motifs (formed on the one hand by TM2 Gly76 and Gly80 and on the other hand by TM11 Gly490 and Gly494), which face each other. The glycine-to-aspartic acid 494 substitution in such an environment is predicted to cause local structure instability, as the p.Gly490Asp substitution which appears to be particularly frequent in patients originating from the Reunion Island and has been associated with a significant reduction of the SLC40A1 cell surface expression [29]. Second, an additional bond (H bond) is formed between the side chain of Arg88 and the oxygen main-chain atom of Leu233, further stabilizing the Arg489-Asp157-Arg88 interaction network. The Leu hydrophobic amino acid, which emerges at the end of a short helix that is located immediately after TM6, also makes CH-𝛑 contacts with Trp158 within the hydrophobic core of the gate. The leucine-to-proline 233 substitution, which has also been proven to reduce the cell surface expression of SLC40A1 [29], is likely to affect the interaction with Trp158 but also, via a modification of the local conformation of the short helix, lead to the loss of the H bond with Arg88. The p.Leu233Val missense mutation might have a more moderate effect, impairing the interaction with Trp158 without necessarily affecting the conformation of the small helix and the H bond with Arg88.

It has previously been established that the thiol group in the side chain of Cys326 in the SLC40A1 C-lobe is mandatory for hepcidin binding [42], and it has recently been demonstrated that the sulfur atom of this particular amino acid interacts with the main-chain oxygen atoms (O and OXT) of hepcidin Thr25 (3.28 and 3.78 Å; Figure 3(b)) [34]. As the functionally characterized and well-defined p.Cys326Ser and p.Cys326Tyr GoF mutants (Table 1), it must therefore be assumed that the p.Cys326Phe missense variant hampers the posttranslational regulation of SLC40A1 by hepcidin.

As shown in Figure 3(b), Tyr64 and Asn144 make part of the hepcidin-binding pocket within the SLC40A1 N-lobe. The Asn144 side chain atoms interact with the hydroxyl atom of Tyr501 (3.4 Å and 3.7 Å with the OD1 and ND2 atoms of Asn144), which stacks with the imidazole side chain of hepcidin His3. Tyr64 is located less deeply in the pocket, interacting with hepcidin Phe4 and Ile6, and it has been suggested that the outward displacement of TM2 near this amino acid pun hepcidin binding plays a key role in SLC40A1 ubiquitination [34]. In support to this idea, the p.Tyr64Asn missense mutation has been consistently reported to reduce SLC40A1 ubiquitination and prevent its intracellular degradation, without altering hepcidin binding [24, 43]. It can be assumed that the p.Tyr64His substitution produces similar effects. Regarding the SLC40A1-Tyr501/hepcidin-His3 interaction, it is worthwhile to remember the results obtained by Aschemeyer et al. showing that the p.Tyr501Cys mutant is no longer able to interact with hepcidin, in a manner comparable to the p.Cys326Ser mutant [24]. Less obvious, perhaps, is the situation of the mutants described at the Asn144 position (p.Asn144His, p.Asn144Asp, and Asn144Thr), which have been investigated by different groups and have been most commonly reported as partial GoF variants with no formal demonstration of a loss of interaction between hepcidin and SLC40A1 (all references provided in Table 1). This is well exemplified by the p.Asn144Asp variant which has been alternatively described as a variant that prevents binding to hepcidin [24] and as a variant that does not (or in a very partial manner [43]), although both studies indicate that SLC40A1 resistance to hepcidin is incomplete.

### 2.4. Performance of *In Silico* Prediction Tools for the Classification of *SLC40A1* Missense Variants

There are a number of software algorithms which can be used to predict the impact of novel missense variants on protein function. In the present study, we evaluated the performance and reliability of ten prediction tools commonly used in medical genetics and by different web-based classification tools, namely, SIFT [44], PolyPhen-2 [45], MutationTaster2 [46], GERP++ [47], MetaLR [48], MetaSVM [48], UMD-Predictor [49], REVEL [50], CADD [51, 52], and DANN [53]. Our observations are summarized in Supplementary Table 2 and Supplementary Figure 2.

As they stand (i.e., considering 48 pathogenic variants and only 8 substitutions recognized as benign), our observations are consistent with a variety of results from similar studies [54–57], each confirming that computational methods generate a low rate of false negative but a high rate of false positive. The combination of two predictive neural networks (CADD and REVEL), as tested for the first time in the present study for *SLC40A1* variants (versus older studies where *in silico* prediction tools were tested separately [20, 23]), did not overcome this drawback. At best, it may help focus resources on variants that could warrant family studies and/or functional explorations.

## 3. The UMD-*SLC40A1* Database

We have established a web-based, manually curated database that gives a complete overview of the *SLC40A1* variants which have been published in the medical literature and have been associated with mild to severe iron overload phenotypes. This database has been developed to aid both clinicians and scientists. It has been constructed from the generic software called UMD (“Universal Mutation Database” [58]), which is today recognized by the Human Genome Organization (HUGO) and the Human Genome Variation Society (HGVS) as a reference tool to build the Locus Specific Databases (LSDB). All the software has been built with the 4^th^ dimensional language (4D). *UMD-SLC40A1* is a dynamic database, in the meaning that it also includes various computerized tools for the analysis of variants. Project and online publishing was approved by the French supervisory authority (“Commission Nationale pour l'Informatique et les Libertés”; registration no. 908361) and the national ethics committee, Comité Consultatif pour le Traitement de l'Information en matière de Recherche dans le domaine de la Santé (no. 07.421), and registered under no. 91513. The database will be freely accessible online at http://umd.be/SLC40A1/.

UMD-*SLC40A1* currently contains the 65 variants presented in this article, which have been split into 336 entries (one per patient). We excluded a few patients harboring the p.Arg88Gly, p.Cys326Ser, p.Asp181Val, p.Gly204Ser, p.His507Arg, or p.Ser209Leu missense mutation due to the lack of individual phenotypic information [30, 59–63]. A unique entry number was created for each patient, and a second number was generated for each family; relatives (*N* = 196) were assigned a number based on the index case (*N* = 140) and the family numbers. Phenotypic data had to include gender, age at diagnosis, transferrin saturation, and serum ferritin. Other clinical data were systematically recorded when available; these included laboratory findings (hemoglobin, hematocrit, red blood cells, mean corpuscular volume, C-reactive protein, *γ*-glutamyl transferase, aspartate transaminase, alanine transaminase, and so on), magnetic resonnance imaging and/or biopsy data (hepatic iron concentration, tissue-specific localization of iron) and clinical manifestation of iron overload (asthenia, arthralgia, skin pigmentation, type 1 or type 2 diabetes mellitus). Patterns of excessive alcohol consumption and the metabolic syndrome, which cause hyperferritinemia, with or without associated iron overload, were also documented. The standard PubMed identifier (PMID) of each publication where phenotypic data were retrieved was entered into the database and linked to the relevant entry numbers. Relatives with negative *SLC40A1* genetic testing were not included in the database.

Each variant was classified using a process consistent with the guidelines of the American College of Medical Genetics and Genomics and the Association for Molecular Pathology in one of the following five classes [64]: benign variant (BV, class 1), likely benign variant (LBV, class 2), variant of uncertain significance (VUS, class 3), likely pathogenic variant (LPV, class 4), and pathogenic variant (PV, class 5). We informed manually the criteria based on our literature review and the GnomAD population database. For familial segregation, we calculated the cosegregation score as described by Jarvik and Browning [65].

Overall, we have described each variant in accordance with its (i) positions in the *SLC40A1* gene (c.) and the human SLC40A1 protein (p.), also considering the protein structure (TM: transmembrane helices and ECL or ICL: extra- or intracellular loops), (ii) reported functional effect (loss-of-function, gain-of-function, and neutral), (iii) ACMG/AMP classification, and (iv) relationship with clinical phenotypes (ferroportin disease, hemochromatosis type 4, and unexplained hyperferritinemia).

## 4. Clinical Relevance of *SLC40A1* Variants and Genotype-Phenotype Correlations

Among the phenotypes associated with *SLC40A1* pathogenic or likely pathogenic mutations, ferroportin disease appears to be the most widely represented, with 29 loss-of-function mutations identified in 227 patients (96 index cases and 131 relatives). Hemochromatosis type 4 is associated with 21 gain-of-function mutations, identified in a total of 81 patients (27 index cases and 54 relatives). Unexplained hyperferritinemia only concerns 35 patients (22 index cases and 13 relatives), 17 of which have a variant that remains classified as a variant of uncertain significance and 18 have a benign or likely benign variant. The clinical characteristics and outcomes of all 343 patients are shown in Supplementary Table 3.

**Figure 4:**
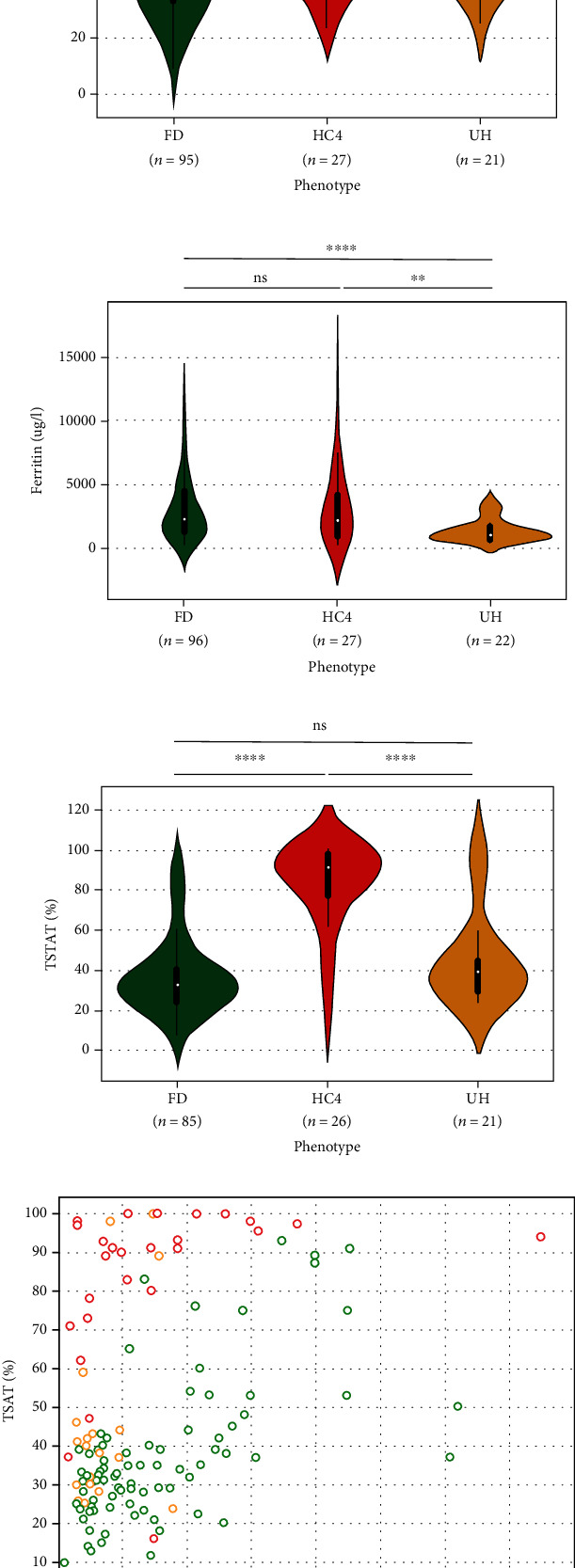
Iron overload profiles of the 96 probands with ferroportin disease (FD), 27 probands with hemochromatosis type 4 (HC4), and 22 probands with unexplained hyperferritinemia (UH). Distributions of age (a), serum ferritin (b), and transferrin saturation (c); medians (white dot on the violin plots) and interquartile ranges (the black bars in the center of violins) are shown. *p* values were calculated on Student's *t*-test; ^∗∗^*p* < 0.01 and ^∗∗∗∗^*p* < 0.0001. (d) Relationship between serum ferritin and transferrin saturation in FD (green), HC4 (red), and UH patients (orange).

FD, HC4, and UH probands were identified at similar ages (Figure 4(a) and Table 2), most often after age 40 (mean, 44.6, 48.7, and 51.7 years, respectively). However, they presented different biological profiles (Figures 4(b) and 4(c)). Serum ferritin concentrations were significantly higher in FD and HC4 patients (median, 2,258 *μ*g/L interquartile range: (1,292; 4,356) and 2,195 *μ*g/L (1,000; 4,326) vs. 1,008 *μ*g/L (777; 1,733); *p* < 0.0001), while transferrin saturation levels were significantly higher in HC4 patients (mean, 83 vs. 37 and 45%; *p* < 0.0001). A fairly clear division also appeared between FD and HC4 probands when transferrin saturation and serum ferritin were matched together (Figure 4(d)).

**Figure 5:**
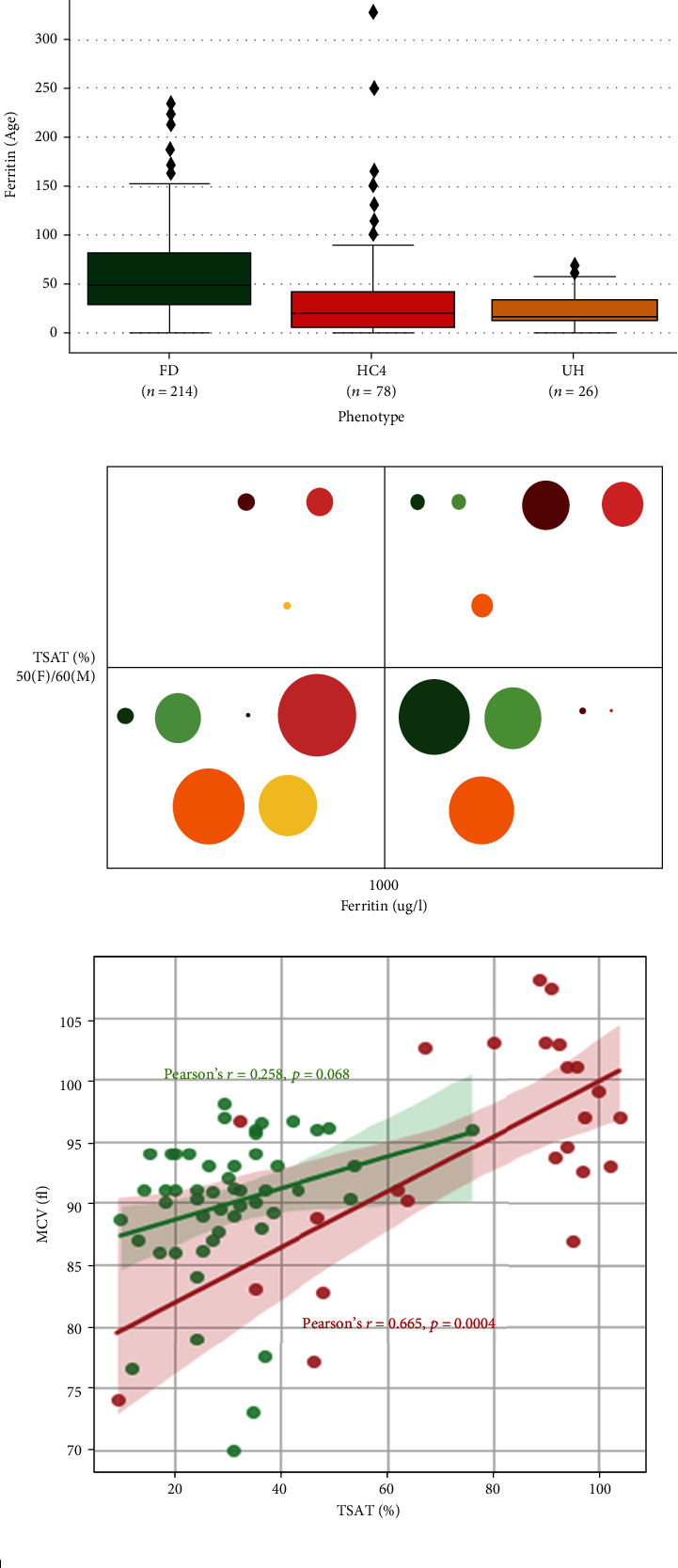
Iron overload profile of the 227 patients with ferroportin disease (FD), 81 patients with hemochromatosis type 4 (HC4), and 35 patients with unexplained hyperferritinemia (UH). (a) Relationship between serum ferritin and age. Linear regression lines with 95% confidence interval have been fitted to the values from FD (green) and HC (red) patients. Pearson's correlation coefficients (*r*) are provided. (b) Box plots show median and range for the ferritin/age ratio. *p* values were calculated on Student's *t*-test; ^∗∗∗∗^*p* < 0.0001. (c) Categorization of the FD (green), HC4 (red), and UH (orange) patients according to serum ferritin and transferrin saturation cutoffs. Dark colors represent the index cases, whereas light colors represent the relatives. Sizes of the circles are proportional to the number of patients. (d) Relationship between mean corpuscular volume and transferrin saturation. Linear regression lines with 95% confidence interval have been fitted to the values from FD (green) and HC (red) patients. Pearson's correlation coefficients (*r*) are provided. (e) Frequency distribution of clinical signs observed in FD (green), HC4 (red), and UH (orange) patients.

FD is characterized by iron accumulation in macrophages, leading to increased production of ferritin, and it releases into plasma. This can occur quite early in life [13] and can continue until very high serum ferritin concentrations that do not necessarily correlate with body iron stores [19]. This is well illustrated in Figure 5(a), which shows a positive correlation between age and serum ferritin in patients with a LoF mutation (*r* = 0.413, *p* < 0001). Further data are provided in Figure 5(b), showing a clear distinction in the ferritin/age ratio between LoF mutation patients and both GoF mutation patients and ACMG classes 1-3 variant patients. At referral (i.e., diagnosis or familial screening), 75% of LoF mutation patients had a ferritin/age ratio ≥ 28.1; this value was reached by only 38% of GoF mutation patients and 20% of patients with an unrecognized pathogenic variant.

HC type 4 is usually associated with parenchymal iron deposition similar to *HFE*-related hemochromatosis and other rare forms of the disease resulting from inadequate levels of serum hepcidin to control systemic iron homeostasis. An elevated transferrin saturation level is considered a sign of early disease, which always precedes an elevated serum ferritin. This is well illustrated in Figure 5(c), which shows a very clear separation of patient groups according to mutation classes. Interestingly, patients with GoF mutations located within the FPN1 C-lobe had higher transferrin saturation values. There was no clear association with age at diagnosis compared with the group of patients with GoF mutations located in the FPN1 N-lobe (Supplementary Figures 3A and 3B). Rather, this could be a consequence of more important hepcidin resistance effects, especially for mutations that affect critical residues (hepcidin and/or iron binding) in transmembrane helices 7 and 11. With a slightly larger data set than that constituted by Zhang et al. in 2018, we confirm another interesting observation, which is the positive correlation between transferrin saturation and size of the red blood cells in GoF mutation patients (Figure 5(d); *r* = 0.665, *p* < 0001). The cellular mechanism leading to this observation is not well understood, but it might be related to the specific function of SLC40A1 in erythroid cells to support hemoglobin production while avoiding iron toxicity and participating in the maintenance of systemic iron homeostasis [5].

Information on clinical manifestation in patients with disease-causing mutations is quite sparse in literature (Figure 5(e)). This is particularly true for patients with a LoF mutation, which may suggest that macrophages are relatively tolerant to iron accumulation. Over time, however, iron can escape into the bloodstream, resulting in increased transferrin saturation as shown in Supplementary Figures 3C and 3D and iron deposition in parenchymal cells. This makes more likely iron-induced oxidative stress and tissue injury [19]. In line with this idea, we observed that 10 of the 16 LoF mutation patients in whom liver fibrosis had been reported also had high transferrin saturation levels (≥ 60% in men and ≥ 50% in women), pointing that three transferrin saturation values were missing in the 16 considered patients. GoF mutation patients had the highest proportions of clinical signs. They also presented with higher aspartate (47 vs. 27 UI/L; *p* < 0.01) and alanine transaminase (83 vs. 31 UI/L; *p* < 0.01) values (Table 2), which is classically considered a sign of hepatocellular injury.

There is a general trend toward mild hyperferritinemia without evidence of plasma iron overload (transferrin saturation level < 50% in females and <60% in males) in patients with variants classified in ACMG categories 1-3 (Figures 4(b)–4(d) and 5(c)), and rather unexpectedly, this trend is more evident in patients with a variant of uncertain significance (Supplementary Figures 3E and 3F). It should be mentioned here that two variants with questionable classification, namely, p.Leu129Pro and p.Ser209Leu, account for a total of three probands and nine relatives (Supplementary Table 4). Iron parameters in the three probands were rather suggestive of FD. However, none of them had a serum ferritin greater than 1000 *μ*g/L in age at diagnosis between 43 and 59 years. Although the penetrance of the different variants associated with FD is thought to be incomplete, it can further be noted that none of the relatives with the p.Leu129Pro had serum ferritin concentrations clearly above the normal range (338 *μ*g/L at most in a 73-year-old male). The p.Ser209Leu variant was studied *in vitro*, but contrary to what the phenotypes of the 7 patients of Asian origin reported in the literature suggest, it was associated with a GoF effect [30]. Also, it is not very rare in the general population (allele frequency in the Genome Aggregation Database V2.1.1 = 0.01%) and more particularly in the eastern region of Asia where its estimating allele frequency reaches 0.06%. New phenotypic data and improved functional analysis would allow to reclassify the two variants, most likely as nonpathogenic.

## 5. Conclusion and Future Prospects

With the increasing description of case reports in the literature, or small family structures with hyperferritinemia, phenotyping is emerging as an important source of uncertainty in the diagnosis of FD and HC4. In such a context, no physician prescribing a genetic test should forget that approximately 90% of patients with mildly elevated serum ferritin do not have iron overload [66, 67], and that magnetic resonance imaging of the abdomen is a very reliable noninvasive tool to confirm iron excess in the liver, spleen, and/or bone marrow and possibly categorize FD and HC4 [68]. On the other hand, no biologist should forget that benign, but rare, missense variants represent a nonnegligible part of human genetic diversity [26] and that this also applies to the *SLC40A1* gene. Less than one-third of the 213 missense variants reported in GnomAD at a frequency < 0.01% are actually suspected to cause iron overload. The “ferroportin score” very recently proposed by Landemaine et al. to increase the proficiency of genetic testing for FD [69] could conceivably also be used to better distinguish pathogenic loss-of-function mutations from neutral rare variants identified by chance in patients with a secondary (but perhaps not clearly defined) cause of hyperferritinemia; we could not test this score because of the piecemeal information available in the literature (only 9 patients for whom the 5 considered criteria could have been reliably used).

We have established a web-based database which gives a comprehensive update of the all uncommon *SLC40A1* coding variants that have been described in literature and individually associated with biological and clinical findings. It will help investigators and clinicians to better distinguish causal mutations from rare polymorphisms. Our database will also allow inclusion of new cases, hoping to contribute something even more determinant in the progression of knowledge and understanding of the molecular bases of FD and HC4. There is considerable variation in the data currently available regarding the onset, progression, and type of clinical manifestations that a patient may develop. The cause of this variability is not fully understood and can only be partially explained by a lack of penetrance, the influence of nongenetic factors, and the description of two categories of causative mutations.

That almost all of the variants described at the *SLC40A1* locus correspond to missense variants undeniably represents an important challenge in the quest for an optimal clinical interpretation, and state-of-the-art prediction tools do not really help. False-negative predictions are rare, and MutationTaster might be judged an appropriate approach for pathogenicity exclusion (at least), but specificity is a recurrent problem that mandates the use of additional data. Interestingly, we have noted that mechanisms associated with the pathogenesis of LoF and GoF missense variants pretty well correlate with particular regions of the SLC40A1 three-dimensional structure. Similar observations have been made in the context of many other genetic diseases [70]. The combination of structural and functional analyses is a promising way to further clarify the SLC40A1 biology and the molecular mechanism of downregulation by hepcidin and, by the way, improve predictions of pathogenicity.

## Figures and Tables

**Table 1:** Comprehensive list of published *SCL40A1* variants.

Exon	Nucleotide change	Amino acid change	Localization in protein	Index cases (*n*=) (PMID)	Functional classification		ACMG/AMP classification	Diagnosis
					*In vitro* findings (PMID)	Structural predictions (this study)		
3	c.190T>A	p.Tyr64Asn	TM2	1 (12857562)	Gain-of-function (15831700, 15692071, 19709084, 19383972, and 23943237)		Pathogenic	Hemochromatosis type 4

3	c.190T>C	p.Tyr64His	TM2	1 (25396007)		Deleterious	Likely pathogenic	Hemochromatosis type 4

3	c.205G>A	p.Ala69Thr	TM2	2 (27177411, 29154924)	Gain-of-function (24859227, 29154924)		Likely pathogenic	Hemochromatosis type 4

3	c.214G>T	p.Val72Phe	TM2	1 (18177470)	Gain-of-function (29237594)		Likely pathogenic	Hemochromatosis type 4

3	c.230C>A	p.Ala77Asp	TM2	3 (10471458, 15951560, and 18160317)	Loss-of-function (15831700, 15692071, 16885049, 23943237, 19846751, 15935710, 23784628, 21396368, 24714983, 24859227, 30002125, and 32450003)		Pathogenic	Ferroportin disease

3	c.238G>A	p.Gly80Ser	TM2	12 (16135412, 16885049, 17052926, 18420432, 21094556, 24370385, and 24714983)	Loss-of-function (21094556, 24714983, and 16885049)		Pathogenic	Ferroportin disease

3	c.239G>T	p.Gly80Val	TM2	1 (16351644)		Deleterious	Likely pathogenic	Ferroportin disease

3	c.252C>G	p.Asp84Glu	TM2	1 (28681497)	Loss-of-function (28681497)		Likely pathogenic	Ferroportin disease

3	c.262A>G	p.Arg88Gly	TM3	3 (17951290, 24714983)	Loss-of-function (23943237, 24714983)		Likely pathogenic	Ferroportin disease

3	c.263G>C	p.Arg88Thr	TM3	1 (16257244)		Deleterious	Pathogenic	Ferroportin disease

3	c.263G>T	p.Arg88Ile	TM3	1 (33385755)		Deleterious	Pathogenic	Ferroportin disease

4	c.386T>C	p.Leu129Pro	TM4	1 (24644245)			VUS	Unexplained hyperferritinemia

5	c.430A>C	p.Asn144His	TM4	1 (11431687)	Gain-of-function (15831700, 15692071, 15956209, 19846751, 15935710, 23784628, 24859227, 19937651, and 30002125)		Pathogenic	Hemochromatosis type 4

5	c.430A>G	p.Asn144Asp	TM4	1 (15030991)	Gain-of-function (15692071, 19383972, and 29237594)		Pathogenic	Hemochromatosis type 4

5	c.431A>C	p.Asn144Thr	TM4	1 (12865285)	Gain-of-function (19383972, 19846751)		Pathogenic	Hemochromatosis type 4

5	c.431A>G	p.Asn144Ser	TM4	1 (18403150)		Deleterious	Likely pathogenic	Hemochromatosis type 4

5	c.454A>T	p.Ile152Phe	TM4	1 (18713659)	Loss-of-function (23784628, 18713659)		Likely pathogenic	Ferroportin disease

5	c.469G>T	p.Asp157Tyr	TM4	1 (24714983)	Loss-of-function (24714983)		Pathogenic	Ferroportin disease

5	c.470A>G	p.Asp157Gly	TM4	3 (12730114, 24714983)	Loss-of-function (15935710, 15956209, 16457665, and 24714983)		Likely pathogenic	Ferroportin disease

5	c.469G>A	p.Asp157Asn	TM4	2 (18177470, 29154924)		Deleterious	Likely pathogenic	Ferroportin disease

5	c.470A>C	p.Asp157Ala	TM4	3 (20230395, 20533066, and 26183747)		Deleterious	Likely pathogenic	Ferroportin disease

5	c.474G>T c.474G>C	p.Trp158Cys	TM4	2 (21396368, 28110135)	Loss-of-function (21396368)		Pathogenic	Ferroportin disease

5	c.479T>C	p.Val160Ala	TM4	1 (34828384)			VUS	Unexplained hyperferritinemia

5	c.484_486del	p.Val162del	TM4	20 (12091366, 12091367, 12123233, 12406098, 15671438, 15753550, 15986403, 17052926, 17997113, 18160317, 24714983, 25742196, 25744502, 29154924, and 31689754)	Loss-of-function (15692071, 15935710, 15956209, 17729390, 28681497, 19846751, 16457665, and 24714983)		Pathogenic	Ferroportin disease

6	c.521A>T	p.Asn174Ile	TM5	3 (16135412, 16885049)	Loss-of-function (24767627, 23784628, 16885049, and 29154924)		Likely pathogenic	Ferroportin disease

6	c.533G>A	p.Arg178Gln	TM5	9 (17997113, 17951290, 30002125, and 32450003)	Loss-of-function (30002125, 32450003)		Pathogenic	Ferroportin disease

6	c.536G>C	p.Arg179Thr	TM5	1 (34828384)			VUS	Unexplained hyperferritinemia

6	c.539T>C	p.Ile180Thr	TM5	3 (16257244, 22890139, and 24714983)	Neutral (24714983)		Likely benign	Unexplained hyperferritinemia

6	c.541G>A	p.Asp181Asn	TM5	1 (29154924)	Loss-of-function (29154924)		Pathogenic	Ferroportin disease

6	c.542A>T	p.Asp181Val	TM5	4 (16351644, 24714983, and 24859227)	Loss-of-function (24767627, 24859227, and 24714983)		Pathogenic	Ferroportin disease

6	c.546G>T	p.Gln182His	TM5	1 (12730114)	Gain-of-function (15935710, 15956209, and 16457665)		Likely pathogenic	Hemochromatosis type 4

6	c.553A>G	p.Asn185Asp	TM5	2 (16111902, 21199650)	Loss-of-function (23943237)		Pathogenic	Ferroportin disease

6	c.610G>C	p.Gly204Arg	TM6	1 (29154924)	Gain-of-function (29154924)		Pathogenic	Hemochromatosis type 4

6	c.610G>A	p.Gly204Ser	TM6	2 (21199650, 21411349)	Gain-of-function (23943237, 29237594)		Pathogenic	Hemochromatosis type 4

6	c.626C>T	p.Ser209Leu	TM6	2 (27896572, 28110135)			VUS	Unexplained hyperferritinemia

6	c.689C>A	p.Thr230Asn	IC3	1 (24714983)	Neutral (24714983)		Likely benign	Unexplained hyperferritinemia

6	c.698T>C	p.Leu233Pro	IC3	2 (18713659, 24714983)	Loss-of-function (18713659, 24714983)		Likely pathogenic	Ferroportin disease

6	c.697C>G	p.Leu233Val	IC3	2 (32360131)		Deleterious	Likely pathogenic	Ferroportin disease

6	c.718A>G	p.Lys240Glu	IC3	1 (21175851)	Gain-of-function (22682227)		Likely pathogenic	Hemochromatosis type 4

6	c.744G>T	p.Gln248His	IC3	4 (14636642, 14636644, and 24714983)	Neutral (15692071, 15831700, 23065513, and 24714983)		Benign	Unexplained hyperferritinemia

7	c.797T>C	p.Met266Thr	IC3	1 (24714983)	Neutral (24714983)		Likely benign	Unexplained hyperferritinemia

7	c.809A>T	p.Asp270Val	IC3	2 (15338274, 22584997)	Gain-of-function (29237594)		Likely pathogenic	Hemochromatosis type 4

7	C.887G>A	p.Arg296Gln	IC3	1 (29154924)	Loss-of-function (29154924)		Likely pathogenic	Ferroportin disease

7	c.968G>T	p.Gly323Val	TM7	1 (12730114)	Loss-of-function (15935710, 15956209, and 16457665)		Likely pathogenic	Ferroportin disease

7	c.977G>C	p.Cys326Ser	TM7	1 (15727899)	Gain-of-function (29237594, 19383972)		Pathogenic	Hemochromatosis type 4

7	c.977G>A	p.Cys326Tyr	TM7	1 (19342478)	Gain-of-function (15692071, 15831700, 23784628, 19937651, 30002125, 32450003, 23065513, 21396368, 24859227, 28681497, and 24714983)		Pathogenic	Hemochromatosis type 4

7	c.977G>T	p.Cys326Phe	TM7	1 (26059880)		Deleterious	Likely pathogenic	Hemochromatosis type 4

7	c.997T>C	p.Tyr333His	TM7	3 (30500107)	Gain-of-function (30500107)		Likely pathogenic	Hemochromatosis type 4

7	c.1014T>G	p.Ser338Arg	TM8	1 (17383046)	Gain-of-function (17383046, 19846751, and 29237594)		Pathogenic	Hemochromatosis type 4

7	c.1035G>C	p.Leu345Phe	TM8	1 (24714983)	Neutral (24714983)		Likely benign	Unexplained hyperferritinemia

7	c.1049C>A	p.Ala350Asp	TM8	1 (34828384)			VUS	Unexplained hyperferritinemia

7	c.1051A>G	p.Ile351Val	TM8	1 (24714983)	Neutral (24714983)		Likely benign	Unexplained hyperferritinemia

7	c.1328C>T	p.Pro443Leu	EC5	1 (24714983)	Neutral (24714983)		Benign	Unexplained hyperferritinemia

7	c.1402G>A	p.Gly468Ser	TM10	1 (18160816)	Loss-of-function (splicing defect) (33341511)		Likely pathogenic	Ferroportin disease

8	c.1467A>C	p.Arg489Ser	TM11	1 (16258219)		Deleterious	Likely pathogenic	Ferroportin disease

8	c.1466G>A	p.Arg489Lys	TM11	1 (19937651)	Loss-of-function (19937651)		Pathogenic	Ferroportin disease

8	c.1468G>A	p.Gly490Ser	TM11	3 (17951290, 24714983)	Loss-of-function (24714983)		Pathogenic	Ferroportin disease

8	c.1469G>A	p.Gly490Asp	TM11	9 (12873829, 24714983)	Loss-of-function (15692071, 15956209, and 24714983)		Likely pathogenic	Ferroportin disease

8	c.1481G>A	p.Gly494Asp	TM11	1 (34828384)		Deleterious	Likely pathogenic^∗^	Ferroportin disease

8	c.1502A>G	p.Tyr501Cys	TM11	1 (19709084)	Gain-of-function (19709084, 24767627, and 29237594)		Likely pathogenic	Hemochromatosis type 4

8	c.1510G>A	p.Asp504Asn	TM11	1 (24714983)	Gain-of-function (24714983, 29237594)		Likely pathogenic	Hemochromatosis type 4

8	c.1520A>G	p.His507Arg	TM11	3 (21396368, 27629970, and 33673803)	Gain-of-function (21396368, 29237594)		Likely pathogenic	Hemochromatosis type 4

8	c.1531G>A	p.Val511Ile	TM11	1 (30500107)			VUS	Unexplained hyperferritinemia

8	c.1592T>C	p.Val531Ala	TM11	1 (34828384)			VUS	Unexplained hyperferritinemia

8	c.1681A>G	p.Arg561Gly	C-ter	2 (19066423, 24714983)	Neutral (24714983)		Benign	Unexplained hyperferritinemia

*SLC40A1* variants are documented based on GenBank accession number NM_014585.5. This table does not include three noncoding variations (c.-181A>G, c.-428-427GG>TT, and c.-59-45del) whose functional impact is unknown and nine missense variations (p.Ala45Glu, p.Ala69Val, p.Ser71Phe, p.Trp158Leu, p.Asn185Thr, p.Ala232Asp, p.Gly267Asp, p.Arg371TRp, and p.Arg371Gln) due to the lack of individual phenotypic information ([71]; [72]; [73]; [21]; [74]). ^∗^Given our predictions in the context of the SLC40A1 3D structure (pdb 6WBV; Figure 3), we propose to use the supporting evidence code PP5 for the p.Gly494Asp variant and classify it in class 4 (instead of class 3); all other variants discussed in Section 2.3 are rated as class 4 or class 5 regardless of the PP5 criteria.

**Table 2:** Clinical and biological data of the 145 index cases.

Index cases						
	FD (*n* = 96)	HC4 (*n* = 27)	UH (*n* = 22)	FD vs. HC4 (*p*)	FD vs. UH (*p*)	HC4 vs. UH (*p*)
Age (years)	*n* = 95	*n* = 27	*n* = 21	0.217	0.053	0.467
Median (min–max)	45 (9-77)	48 (24-72)	53 (25-74)			
Gender (*n*)	*n* = 95	*n* = 27	*n* = 22	0.112	0.144	1
Female	39	6	5			
Male	56	21	17			
Ferritin (*μ*g/L)	*n* = 96	*n* = 27	*n* = 22	0.811	<0.0001	0.0085
Median (min–max)	2258 (255-12405)	2195 (353-15000)	1008 (585-3600)			
Transferrin saturation (%)	*n* = 85	*n* = 26	*n* = 21	<0.0001	0.193	<0.0001
Median (min–max)	33 (7-93)	91 (16-100)	39.4 (23.7-100)			
HIC (*μ*mol/g)	*n* = 30	*n* = 6	*n* = 4	0.748	0.786	0.695
Median (min–max)	215 (20-2920)	265.35 (85-925)	170 (106-693)			
Hb (g/dL)	*n* = 56	*n* = 12	*n* = 13	0.383	0.608	0.799
Median (min–max)	14.1 (10.9-17)	14.9 (7.5-16.5)	14.5 (9.5-16.1)			
MCV (fL)	*n* = 34	*n* = 11	*n* = 7	0.007	0.628	0.028
Median (min–max)	91 (70-97)	96.9 (87-108)	90.5 (78.3-101)			
ASAT (UI/L)	*n* = 33	*n* = 14	*n* = 7	0.003	0.201	0.476
Median (min–max)	27 (13-50)	47 (1-322.1)	40 (15-145)			
ALAT (UI/L)	*n* = 36	*n* = 15	*n* = 7	0.002	0.545	0.087
Median (min–max)	31 (11-114)	83 (14-538.8)	48 (14-84)			
GGT (UI/L)	*n* = 24	*n* = 10	*n* = 6	0.145	0.067	0.655
Median (min–max)	22.5 (10-61)	23 (6-345)	42.5 (16.6-582)			
Tissue iron deposition (*n*)						
Hepatocyte	0	6	1			
Macrophage	20	0	0			
Mixed	23	10				

FD = ferroportin disease; HC4 = hemochromatosis type 4; UH = unexplained hyperferritinemia.

## Data Availability

All information about referenced variants is recorded in a locus-specific online database that is accessible at http://umd.be/SLC40A1/. All other data are available upon request.

## Supplementary Materials

Supplementary Materials Supplementary Figure S1: multiple species sequence alignment of ferroportin. Supplementary Figure S2: scatter plot visualisation of CADD and REVEL scores for pathogenic variants, neutral variants, and variants of unknown significance. Supplementary Figure S3: iron overload profiles of the patients with hemochromatosis type 4, ferroportin disease, or unexplained hyperferritinemia. Supplementary Table S1: Grantham scores for the 10 amino acid changes without functional data. Supplementary Table S2: comparison of *in silico* predictors in a subset of 56 *SLC40A1* missense variants (provided as an Excel file). Supplementary Table S3: clinical and biological data of the 343 patients reported in the literature. Supplementary Table S4: clinical, biological data of the 17 patients with functionally unexplored variants.
