# Supplementary material for: The Spectra of Disease-Causing Mutations in the Ferroportin 1 (SLC40A1) Encoding Gene and Related Iron Overload Phenotypes (Hemochromatosis Type 4 and Ferroportin Disease)
Source: Hum Mutat. 2023 Jun 13;2023:5162256. doi: 10.1155/2023/5162256 (PMC11919020; doi:10.1155/2023/5162256)
Supplement: Supplementary Materials — Supplementary Figure S1: multiple species sequence alignment of ferroportin. Supplementary Figure S2: scatter plot visualisation of CADD and REVEL scores for pathogenic variants, neutral variants, and variants of unknown significance. Supplementary Figure S3: iron overload profiles of the patients with hemochromatosis type 4, ferroportin disease, or unexplained hyperferritinemia. Supplementary Table S1: Grantham scores for the 10 amino acid changes without functional data. Supplementary Table S2: comparison of in silico predictors in a subset of 56 SLC40A1 missense variants (provided as an Excel file). Supplementary Table S3: clinical and biological data of the 343 patients reported in the literature. Supplementary Table S4: clinical, biological data of the 17 patients with functionally unexplored variants. [file 5162256.f1.docx]

**Supplementary data**

**Supplementary Figure 1**. **Multiple species sequence alignment of ferroportin.** Residues that correspond to missense variations that have been analyzed *in vitro* and recognized as either LoF or GoF mutations in humans are boxed in grey, whereas residues that correspond to substitutions with no functional data are boxed in orange.


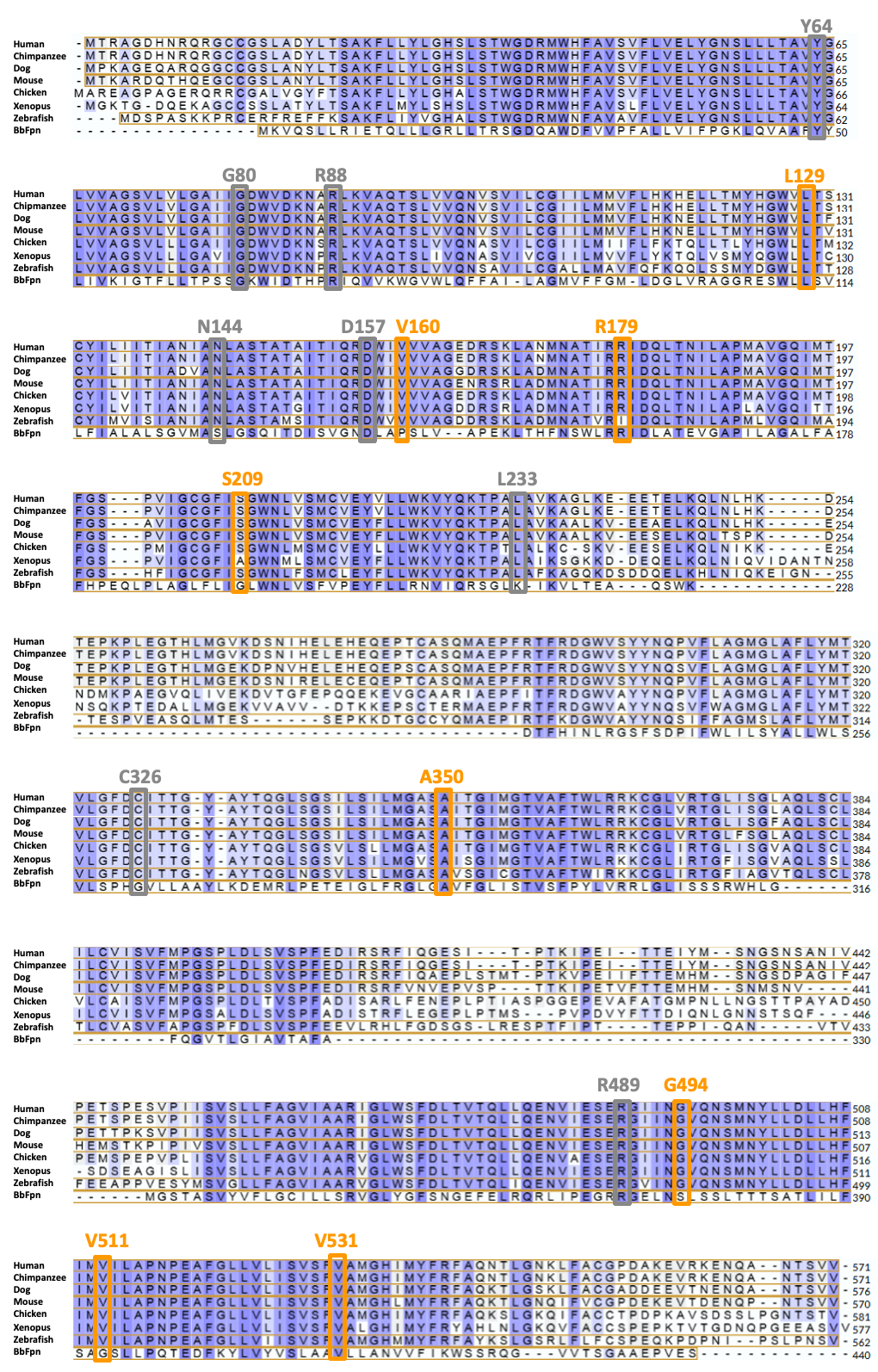


**Supplementary Table 1. Grantham scores (GS) for the 10 amino acid changes without functional data.** The GS ranged from 23 to 205 (corresponding to an exchange between two amino acids separated by a very small physiochemical distance, and a very important physiochemical distance, respectively). Six substitutions were predicted to be conservative (p.Asp157Asn, p.Asn144Ser, p.Leu233Val; GS: 0-50) or moderately conservative (p.Tyr64His, p.Arg88Thr, p.Arg88Ile; GS: 51-100) with lower GS scores than substitutions functionally described as pathogenic (*e.g.* p.Asp157Tyr, p.Asp157Gly, p.Asn144His, p.Asn144Thr, p.Leu233Pro, p.Tyr64Asn, p.Arg88Gly). Conversely, four substitutions were classified as moderately radical (p.Gly80Val, p.Asp157Ala, p.Arg489Ser; GS: 101-150) or radical (p.Cys326Phe; GS >150) with higher GS scores than substitutions functionally described as pathogenic (*e.g.* p. Gly80Ser, Asp157Gly, p.Arg489Lys, p.Cys326Ser, p.Cys326Tyr).

| **Not functionally tested** | | **Functionally tested** | |
| --- | --- | --- | --- |
| **Variant** | **GS** | **Variant** | **GS** |
| p.Tyr64His | 83 | p.Tyr64Asn | 143 |
| p.Gly80Val | 109 | p.Gly80Ser | 56 |
| p.Arg88Thr | 71 | p.Arg88Gly | 125 |
| p.Arg88Ile | 97 |  |  |
| p.Asn144Ser | 46 | p.Asn144His | 68 |
|  |  | p.Asn144Thr | 65 |
| p.Asp157Ala | 126 | p.Asp157Tyr | 160 |
| p.Asp157Asn | 23 | p.Asp157Gly | 94 |
| p.Leu233Val | 32 | p.Leu233Pro | 98 |
| p.Cys326Phe | 205 | p.Cys326Ser | 112 |
|  |  | p.Cys326Tyr | 194 |
| p.Arg489Ser | 110 | p.Arg489Lys | 26 |

**Supplementary Table 2**. **Comparison of *in silico* predictors in a subset of 56 *SLC40A1* missense variants.**

**
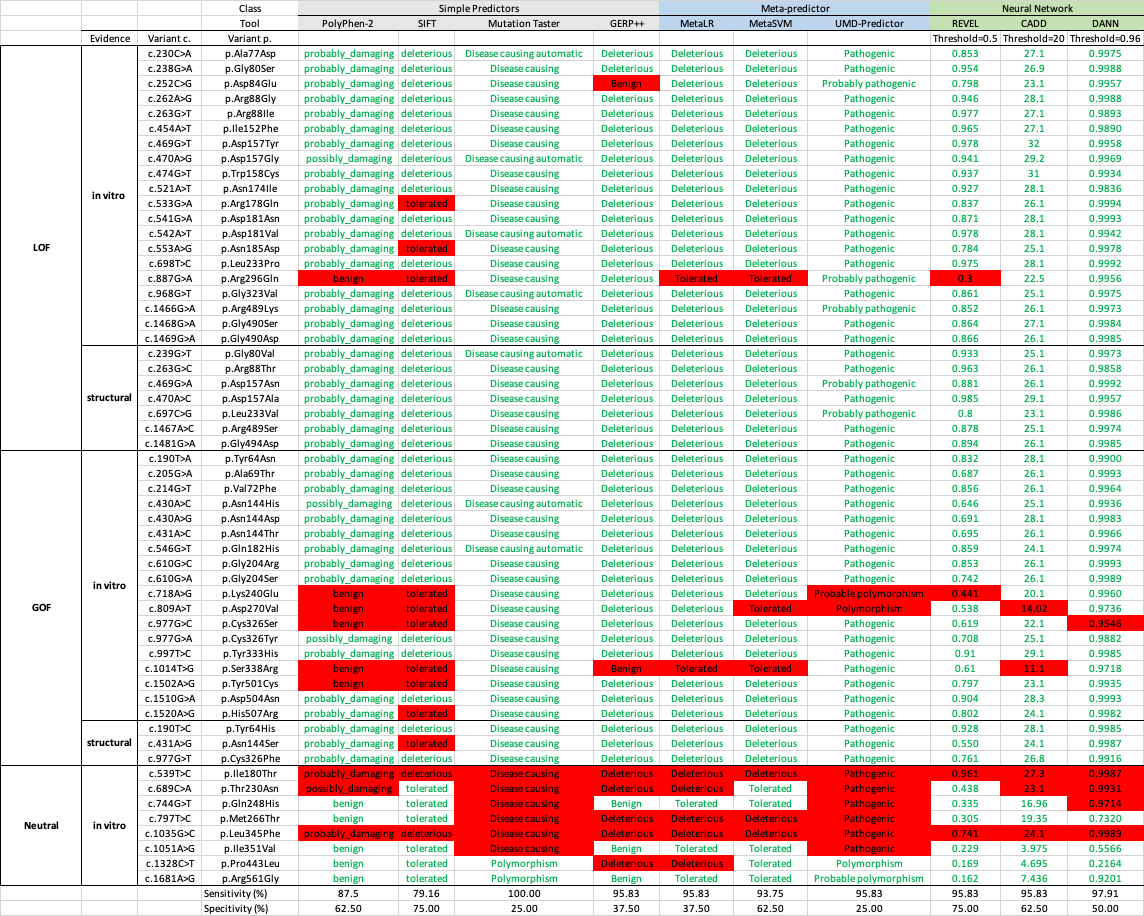
**

**Supplementary Figure 2**. Scatter plot visualisation of CADD and REVEL scores for pathogenic variants (red), neutral variants (blue) and variants of unknown significance (orange). Dot lines represent the cutoffs for REVEL (horizontal) and CADD (vertical) scores.


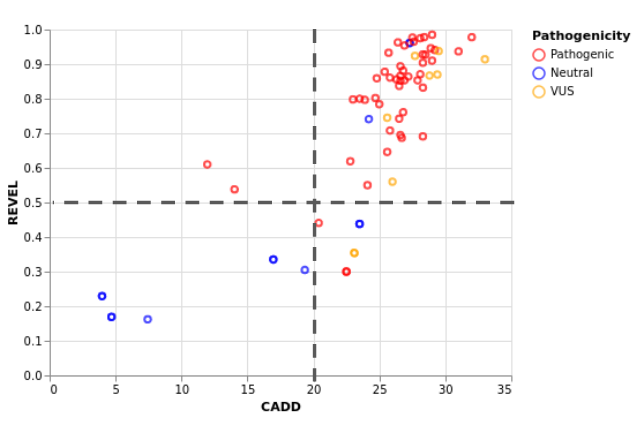


*Comments on Supplementary Table 2 and Supplementary Figure 2:*

Our evaluation set included 27 LoF variants (20 annotated on the basis of experimental data and 7 annotated on the basis of structural analysis), 21 GoF variants (18 annotated on the basis of experimental data and 3 annotated on the basis of structural analysis) and 8 neutrals variants (the only ones for which functional data have been published to date; (Callebaut et al., 2014). By comparing the predictions made by stand-alone tools, we identified Mutation Taster as the only computational approach that did not result in false negative predictions (Supplementary Table 2). We found four LoF missense variant and seven GOF missense variants that were wrongly categorized as neutral by at least one of the other nine *in silico* tools; SIFT having the lowest sensitivity (79.6%). All tools suffered from a lack of specificity, resulting in an excessive rate of variants classified as pathogenic at the forefront of which is Mutation Taster. Seven neutral variants were thus wrongly classified by at least two computational predictors. Two of these variants (p.Ile180Thr and p.Leu345Phe) were misclassified by all ten tools.

By combining the predictions made by two neural networks, namely CADD and REVEL, we reached a positive predictive value (PPV) of 95,7% (44 true positive variants *versus* 2 false positive variants). It is important to note that the four p.Asp270Val, p.Ser338Arg, p.Lys140Glu and p.Arg296Gln amino acid changes were not included in the PPV calculation because they were correctly classified by only one of the two selected tools (Supplementary Figure 2). It also important to emphasize that the two p.Ile180Thr and p.Leu345Phe neutral variants were still wrongly categorized as pathogenic by CADD and REVEL. The sensitivity of the combined approach was 100% (CADD: 95.8%; REVEL: 97.9%), and the specificity was 71.4% (CADD: 62.5%; REVEL: 75.0%); still only considering the 52 (of 57; 91,1%) *SLC40A1* missense variants that did not show conflicting predictions.

The fact that the p.Ile180Thr and p.Leu345Phe variants are systematically classified as pathogenic by the prediction tools may seem curious. Indeed, the two residues are well conserved between species, with the exception of the leucine residue that is changed by a phenylalanine in the primary sequence of the bacterial homologue BpFPN. Furthermore, both residues are located on the hydrophobic side of transmembrane helices (TM5 and TM8, respectively). The hydrophobic character is, however, conserved at these two positions for the observed missense variations, which should therefore not be structurally damaging. Another possible explanation is that the two variants were overlooked due to inappropriate *in vitro* methodologies. It is advisable to remember here that the ACMG/AMP framework assigns strong weight to *in vitro* findings, but not stand-alone (Richards et al., 2015), owing that the biological complexity of a protein cannot be fully reproduced in cellular models.

A major limitation of this evaluation of *in silico* prediction tools is the low proportion of missense variants recognized as benign. An ideal dataset does not have to be composed of dozens of variants (Brnich et al., 2019), but it should always have as many true positives as true negatives.

**Supplementary Figure 3**. **Iron overload profiles of the patients with hemochromatosis type 4 (HC4; A, B), ferroportin disease (FD; C,D) or unexplained hyperferritinemia (UH; E, F).** Box plots show median and range of the transferrin saturation (TSAT) (A) and age (B) values for patients with HC4, according to the location of the variant in the N-terminus lobe (Nter) or C-terminus lobe (Cter) of FPN1. (C, D) Relationship between TSAT and age (C) or ferritin (D) in FD patients. Linear regression lines with 95% confidence interval have been fitted to the values from FD patients. The Pearson’s correlation coefficients (r) are provided. (E, F) Violin plots show the values of TSAT (E) and ferritin (F) in UH patients according to the classification of the variant. VUS=variant of uncertain significance. LB_B= likely benign and benign variants. P values were calculated on Student t-test; *P<0.05, ***P<0.001.


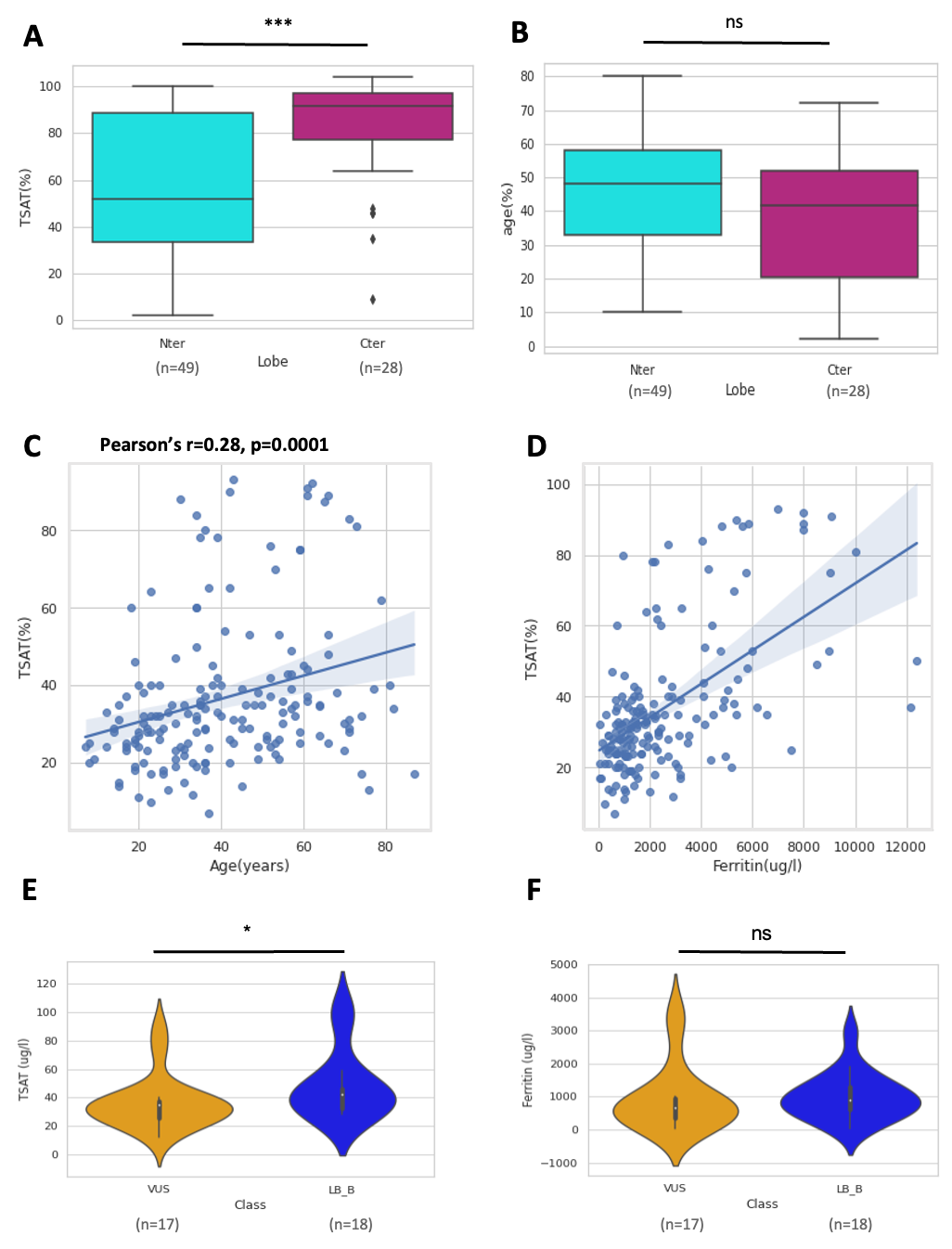


**Supplementary Table 3**. Clinical and biological data of the patients. FD= ferroportin disease, HC4= hemochromatosis type 4, UH= unexplained hyperferritinemia, HIC= hepatic iron concentration, MCV= mean corpuscular volume, Hb=hemoglobin, ASAT= aspartate aminotransferase, ALAT= alanine aminotransferase, GGT= gamma glutamyl transpeptidase.

|  | **FD**  **(n=227)** | **HC4**  **(n=81)** | **UH**  **(n=35)** | | **FD vs HC4**  **p** | **FD vs UH**  **p** | **HC4 vs UH**  **p** |
| --- | --- | --- | --- | --- | --- | --- | --- |
| **Age (years)**  **Median (min-max)** | n=214  37 (6-87) | n=78  46.5 (2-80) | | n=26  50.5 (7-74) | 0.124 | **0.003** | 0.09 |
| **Gender (n)**  **Female**  **Male** | n=227  91  136 | n=79  29  50 | | n=35  11  25 | 0.511 | 0.358 | 0.677 |
| **Ferritin (µg/l)**  **Median (min-max)** | n=227  1600 (38-12405) | n=81  750 (4-15000) | | n=35  719 (23-3600) | **<0.0001** | **<0.0001** | 0.842 |
| **Transferrin saturation (%)**  **Median (min-max)** | n=184  32 (7-93) | n=80  79 (2-104) | | n=30  36 (12-100) | **<0.0001** | 0.065 | **<0.0001** |
| **HIC (µmol/g)**  **Median (min-max)** | n=52  205 (20-2920) | n=9  210.7 (85-925) | | n=4  170 (106-693) | 0.55 | 0.969 | 0.629 |
| **Hb (g/dl)**  **Median (min-max)** | n=106  14.1 (10.9-18.4) | n=29  14.5 (7.5-16.5) | | n=18  14.4 (9.5-16.7) | 0.196 | 0.407 | 0.841 |
| **MCV (fl)**  **Median (min-max)** | n=57  91 (70-98) | n=24  95.6 (74-108) | | n=7  90.5 (78.3-101) | **0.005** | 0.579 | 0.107 |
| **ASAT (UI/l)**  **Median (min-max)** | n=49  25 (12-54) | n=29  32.5 (14-322.1) | | n=7  40 (15-145) | **0.026** | 0.107 | 0.99 |
| **ALAT (UI/l)**  **Median (min-max)** | n=54  30 (6-114) | n=31  62 (14-538.8) | | n=7  48 (14-84) | **0.012** | 0.414 | 0.19 |
| **GGT (UI/l)**  **Median (min-max)**    **Tissue iron deposition (n)**  **Hepatocyte**  **Macrophage**  **Mixed** | n=41  23 (8-61)      0  26  40 | n=21  23 (6-345)      8  0  11 | | n=6  42.5 (16.6-582)      1  0  4 | 0.539 | 0.053 | 0.17 |

**Supplementary Table 4.** Clinical, biological data of the patients with unexplored variants, classified as variants of uncertain significance. For each variant, index cases and relatives are described, as well as the frequency of the variation in the population database gnomAD (v2.1.1.). PMIDs where the patients were reported are referenced.

| Variant c. | Variant p. | gnomAD v2.1.1 frequency | Sex | Age | Relationship | Ferritin (ug/l) | TSAT (%) | PMID |
| --- | --- | --- | --- | --- | --- | --- | --- | --- |
| c.386T>C | p.Leu129Pro | 0 | M | 43 | Index case | 817 | 40 | 24644245 |
|  |  |  | M | 73 | Father | 338 | 37 |  |
|  |  |  | F | 42 | Sister | 24 | 29 |  |
|  |  |  | M | 41 | Brother | 151 | 73 |  |
|  |  |  | M | 7 | Son | 97 | 35 |  |
| c.626C>T | p.Ser209Leu | All population: AC: 42 AF: 1.5.10^-4^  East Asian: AC:13 AF: 6.5.40^-4^ | M | 46 | Index case | 660 | 25.6 | 27896572 |
|  |  |  | F | 67 | Mother | 303 | 25.2 |  |
|  |  |  | M | 48 | Index case | 834 | 25 | 28110135 |
|  |  |  | M |  | Father | 692 | 35 |  |
|  |  |  | M |  | Brother | 359 | 21 |  |
|  |  |  | M |  | son | 544 | 35 |  |
|  |  |  | M |  | son | 576 | 12 |  |
| c.1531G>A | p.Val511Ile | 0 | M | 47 | Index case | 3168 | 89 | 30500107 |
| c.479T>C | p.Val160Ala | 0 | M | 25 | Index case | 1000 | 31.7 | 34828384 |
| c.536G>C | p.Arg179Thr | All population: AC:1 AF: 3.9810^-6^ | M | 41 | Index case | 819 | 39.4 | 34828384 |
| c.1049C>A | p.Ala350Asp | 0 | M | 62 | Index case | 3600 | 23.7 | 34828384 |
| c.1592T>C | p.Val531Ala | 0 | M | 54 | Index case | 1908 | 37 | 34828384 |

**REFERENCES**

Brnich SE, Abou Tayoun AN, Couch FJ, Cutting GR, Greenblatt MS, Heinen CD, Kanavy DM, Luo X, McNulty SM, Starita LM, Tavtigian SV, Wright MW, et al. 2019. Recommendations for application of the functional evidence PS3/BS3 criterion using the ACMG/AMP sequence variant interpretation framework. Genome Med 12:3.

Callebaut I, Joubrel R, Pissard S, Kannengiesser C, Gérolami V, Ged C, Cadet E, Cartault F, Ka C, Gourlaouen I, Gourhant L, Oudin C, et al. 2014. Comprehensive functional annotation of 18 missense mutations found in suspected hemochromatosis type 4 patients. Hum Mol Genet 23:4479–4490.

Richards S, Aziz N, Bale S, Bick D, Das S, Gastier-Foster J, Grody WW, Hegde M, Lyon E, Spector E, Voelkerding K, Rehm HL, et al. 2015. Standards and guidelines for the interpretation of sequence variants: a joint consensus recommendation of the American College of Medical Genetics and Genomics and the Association for Molecular Pathology. Genet Med Off J Am Coll Med Genet 17:405–424.
